# Experience of the temporary discharge from the inpatient palliative care unit: A nationwide post-bereavement survey for end-of-life cancer patients

**DOI:** 10.1016/j.apjon.2022.03.010

**Published:** 2022-04-11

**Authors:** Go Sekimoto, Sakiko Aso, Naoko Hayashi, Keiko Tamura, Chieko Yamamoto, Maho Aoyama, Tatsuya Morita, Yoshiyuki Kizawa, Satoru Tsuneto, Yasuo Shima, Mitsunori Miyashita

**Affiliations:** a Sekimoto Clinic, Kobe, Japan; b Department of Nursing, Shizuoka Cancer Center, Shizuoka, Japan; c Department of Oncology Nursing and Palliative Care, Graduate School of Nursing Science, St Luke's International University, Tokyo, Japan; d Department of Geriatric and Palliative Nursing, Human Health Sciences, Graduate School of Medicine, Kyoto University, Kyoto, Japan; e Kasugai Rehabilitation Hospital, Aichi, Japan; f Department of Palliative Nursing, Health Sciences, Tohoku University Graduate School of Medicine, Sendai, Japan; g Palliative and Supportive Care Division, Seirei Mikatahara Hospital, Shizuoka, Japan; h Department of Palliative and Supportive Care, University of Tsukuba Hospital, Ibaraki, Japan; i Department of Human Health Sciences, Graduate School of Medicine, Kyoto University, Kyoto, Japan; j Tsukuba Medical Center Hospital, Department of Palliative Medicine, Ibaraki, Japan

**Keywords:** Discharge, Inpatient palliative care unit, Neoplasm, Palliative care, Post-bereavement survey

## Abstract

**Objective:**

Inpatient palliative care units (PCUs) have two roles: place of death and symptom control. In case of symptom control, most patients whose distressing symptoms could be relieved would be temporarily discharged back home. However, the experience of the patient and their family during temporary discharge is unclear.

**Methods:**

This study is a part of the Japan HOspice and Palliative Care Evaluation Study 3, a nationwide cross-sectional post-bereavement survey. We sent questionnaires to bereaved relatives of cancer patients who died in PCUs in 2018.

**Results:**

Among 968 questionnaires sent, 571 questionnaires were analyzed (59%). Sixteen percent of patients experienced temporary discharge from PCUs. Seventy-two percent of bereaved family members reported that patients said “I am happy to be discharged home.” Overall, 22%–37% of participants reported improvement in the patient's condition after discharge. The caregiver's recognition of better patient's quality of life at home and the doctor's assurance of re-hospitalization, if necessary, were significantly associated with positive experience.

**Conclusions:**

Bereaved family members recognized temporal discharge as positive experiences for patients and families. Appropriate home palliative care and discharge planning would contribute to positive experience after discharge.

## Introduction

Japanese specialized palliative care started with the incorporation of inpatient palliative care units (PCUs) into the national medical insurance system,^1^ and 13% of cancer deaths occurred in PCUs in 2014.^2^ Japanese PCU has two roles: place of death and symptom control.^3^ In case of symptom control, the patient whose distressing symptoms could be relieved would be discharged back home or transferred to other facilities. In fact, 16% of the patients admitted in PCUs were discharged alive^2^; however, it may be estimated to be lower than reported by acute PCUs in US and Canada.^4^^,^^5^ Another nationwide survey showed that 72% of the patients who left PCU were cared for at home, followed by acute hospital (12%) and care facility (6%), and 39% of them died at home, followed by the PCU (39%) and the acute hospital (15%).^6^ These results suggest that 39% of the discharge were temporal, and required re-admission to PCU again because of worsening symptoms or increased care burden at home.

In contrast, many Japanese patients prefer staying at home during the terminal phase,^7^^,^^8^ and patients and families prefer home care even if re-admission is anticipated because of worsening symptoms or care burden. Therefore, the Japanese health authority obliged PCU to ensure coordinating discharge, cooperating with community-based medical institutions to deliver home care, and accepting the emergent admission of the patient under home care.^9^

Although home is the most commonly preferred place of care worldwide for terminal patients,^10^ positive and negative perspectives and benefits of home care have been reported.^11^^,^^12^ In addition, several post-bereavement surveys reported home death results in achieving good death in Japan.^13^^,^^14^ However, most of these findings are limited to home death, and experiences of patients temporarily discharged from PCU, who were re-admitted and died in PCU have not been reported. Although clinicians have experienced the benefits of temporary discharge empirically, we have no evidence of these benefits.

Therefore, we explored the experiences of patients who were temporarily discharged from PCU from the perspective of the bereaved family members.

## Methods

This study is a part of the nationwide cross-sectional survey for bereaved family members of cancer patients that aims to evaluate the quality of end-of-life care in Japan (the Japan HOspice and Palliative Care Evaluation Study 3: J-HOPE3) and was conducted in 2018.^15^ The -HOPE3 study was a multicenter questionnaire survey of bereaved family members of cancer patients who died in PCU. Overall, 133 PCUs participated in the study nationwide. As part of the J-HOPE3 study, we mailed a questionnaire with two sections to each potential participant from each participating PCU. The first section consisted of common questions for main outcomes such as the evaluation of care. The second section consisted of specific clinical research questions randomly assigned to the potential participants. This article had a specific research question regarding “temporary discharge from 10.13039/501100002599PCU.” The details of the study design were described in the protocol paper.^15^ Ethical approval for the study was granted by the Institutional Review Boards of the 10.13039/501100006004Tohoku University (Approval No. 2013-1-334) and all participating institutions.

### Participants and procedures

Among 13,584 potential participants in the J-HOPE3 study from 133 PCUs, we randomly enroled 968 bereaved family members of cancer patients who died in this study. As part of the J-HOPE3 study, we asked each institution to identify and list up to 80 bereaved family members of patients who had died prior to October 2012 to identify potential participants. The inclusion criteria were as follows: (1) the patient died of cancer, (2) the patient was aged 20 years or older, (3) the bereaved family member was aged 20 years or older, and (4) the duration of the last hospitalization was three days or more. The exclusion criteria were as follows: (1) the bereaved family members could not be identified, (2) the death was associated with treatment, (3) the participant suffered serious psychological distress, as determined by the primary physician, and (4) the participant was incapable of completing the self-reported questionnaire because of health issues such as cognitive impairment or visual disability.

The PCUs where patients were hospitalized and died sent the questionnaire to bereaved family members between May and July 2014. Simultaneously, we extracted medical data of the patients from medical records in PCUs where the patients died. A ballpoint pen was included in the envelope as an incentive to participate. We asked the potential participant to return a completed questionnaire to the secretariat office (Tohoku University) within 2 weeks. The return of the questionnaire was considered as consent to participate in the study. We sent a reminder to non-responders 1 month after sending the questionnaire.

### Questionnaires

#### Experience of discharge reported by the patient to the bereaved family

We asked the bereaved family members whether the patients talked to them about how good or bad it was that he or she was discharged back home; whether the patient said “I am happy to be discharged to home,” “I regret to be discharged to home,” or “both.”

#### Family member's perception of the experiences of temporary discharge

We asked bereaved family members their perception about temporary discharge, patient condition compared to being hospitalized, and family condition compared to the patient being hospitalized.

We developed 17 questions based on literature review,^11^^,^^12^^,^^16^ interview with 10 bereaved family members, and discussion among researchers, and we asked them to rate the experience using a 5-point Likert scale (1: strongly disagree, 2: disagree, 3: unsure, 4: agree, and 5: strongly agree).

#### Circumstances of the patient and family caregiver before and after temporary discharge

We asked the bereaved family members regarding the circumstances of the patient and family caregiver before and after temporary discharge; for example, preference of patient and family members for discharging home, physical, mental, and social status of patient and family before discharge, and consultation and support by health care professionals before and after discharge. We developed 26 questions based on literature review,^11^^,^^12^^,^^16^ interview with 10 bereaved family members and discussion among researchers, and we asked the participants to respond by 1: agree or 2: disagree.

### Participant characteristics

We extracted data regarding the patient's age, gender, primary cancer site, and duration of last hospital stay from the medical record. We asked the bereaved family members their age, gender, relationship with the patient, frequency attending the patient during the last hospitalization, the health status of the family members during the last hospitalization, and end-of-life discussions with the physician and the patient through the questionnaire.

### Data analysis

We compared the characteristics of the patients and bereaved family members between the discharged and no discharged group using Chi-square test. Secondly, for the patients who discharged home temporarily, we conducted descriptive statistics about the experience after temporary discharge and the circumstances of the patient and family caregiver before and after temporary discharge. Lastly, we explored the associations between the positive experience of discharge by patient talk, namely “I am happy to be discharged back home” and the experiences after temporary discharge and circumstances of patient and family caregiver before and after temporary discharge by Chi-square test or Fisher's exact test, as appropriate. All statistical tests were two-tailed with a significance level of 0.05, and all analyses were conducted by SPSS software (Ver.25.0; IBM, Tokyo, Japan).

## Results

Among 968 questionnaires were mailed to bereaved family members of cancer patients, 711 returned. Among them, Seventy-four participants refused to answer, 25 were excluded because of violation of the inclusion criteria, and 41 did not answer the presence or absence of temporary discharge from PCU. Therefore, 571 questionnaires were analyzed (59.0%).

Among 571 people, 90 (15.8%) answered that the patient had experienced temporary discharge from PCU. Duration from discharge back home to re-admission to PCU were as follows; 25 patients (25.6%) stayed at home less for than 3 days, nine (10.0%) for 4–6 days, 11 (12.2%) for 7–13 days, 16 (17.8%) for 14–29 days, and 29 (32.2%) for more than 30 days.

Table 1 shows the participant characteristics between discharge and no discharged group. Gender (Male: *P* ​= ​0.05), longer disease duration of cancer (*P* ​= ​0.004), better mental health status of caregivers during the last hospitalization (*P* ​= ​0.04), frequent attendance of the family members during the last hospitalization (*P* ​= ​0.01) were associated with the discharge.

**Table 1:** Participant characteristics.

	Total	No discharge		Discharged		*P* value
	*N* ​= ​571	*N* ​= ​481		*N* ​= ​90		
	*n*	*n*	％	*n*	％	
Patient						
Age (years), Mean ​± ​SD		73.7 ​± ​11.3		74.5 ​± ​10.6		0.54
Gender						
Male	320	278	59.5	43	48.3	0.05
Female	234	189	40.5	46	51.7	
Primary cancer site						
Lung	147	128	26.7	19	21.1	0.92
Stomach	66	54	11.3	12	13.3	
Colorectum/rectum	64	52	10.8	12	13.3	
Pancreas	58	48	10.0	11	12.2	
Urinary	41	35	7.3	6	6.7	
Liver	28	26	5.4	2	2.2	
Gynecological	29	24	5.0	5	5.6	
Breast	26	22	4.6	4	4.4	
Gall bladder/bile duct	26	21	4.4	5	5.6	
Other	83	70	14.6	14	15.6	
Marital status						
Married	351	294	62.3	59	68.6	0.71
Divorced/widowed	176	152	32.2	24	27.9	
Not married	29	26	5.5	3	3.5	
Disease duration of cancer						
< 3 months	74	73	15.2	1	1.1	0.004
≥ 3 months, < 1 year	157	130	27.1	29	32.6	
≥ 1 year, < 3 years	175	146	30.5	29	32.6	
≥ 3 years	160	130	27.1	30	33.7	
Duration of the last PCU stay (days), Mean ​± ​SD		40.75 ​± ​53.31		34.72 ​± ​32.46		0.96
Preference regarding the place of death (patient)						
Home	221	183	38.6	39	43.3	0.47
PCU	212	177	37.3	35	38.9	
Hospital	25	20	4.2	6	6.7	
Other	3	3	0.6	0	0.0	
No preference	19	17	3.6	2	2.2	
Unsure	82	74	15.6	8	8.9	
Bereaved family member						
Age (years), Mean ​± ​SD		60.84 ​± ​12.08		62.2 ​± ​11.5		0.66
Sex						
Male	174	141	29.9	35	39.3	0.08
Female	385	331	70.1	54	60.7	
Relationship to patients						
Spouse	258	217	45.5	43	47.8	0.22
Child	216	178	37.3	38	42.2	
Others	91	82	17.191	9	10.0	
Physical status during the last hospitalization						
Good	113	87	18.2	26	29.2	0.12
Moderate	315	272	57.0	44	49.4	
Bad	119	104	21.8	16	18.0	
Very bad	17	14	2.9	3	3.4	
Mental status during the last hospitalization						
Good	47	35	7.4	12	13.3	0.04
Moderate	277	227	47.9	51	56.7	
Bad	207	185	39.0	23	25.6	
Very bad	31	27	5.7	4	4.4	
Frequency of attending to the patient (days/week)						
Everyday	369	322	67.1	48	53.3	0.01
4–6	77	60	12.5	17	18.9	
1–3	86	70	14.6	16	17.8	
<1	36	28	5.8	9	10.0	
Preference regarding the place of death (Family)						
Home	129	103	21.8	26	29.5	0.62
PCU	370	314	66.4	56	63.6	
Hospital	25	22	4.7	3	3.4	
Other	2	2	0.4	0	0.0	
No preference	17	15	3.2	2	2.3	
Unsure	18	17	3.6	1	1.1	

PCU, Palliative care unit.

As for the experience of discharge reported by the patient talking to bereaved family, 72% of patients reported: “I am happy to be discharged back home,” 1% reported, “I regret to be discharged back home,” 4% reported “both,” and 20% did not say anything about that.

We showed the perception of the family members regarding the experiences of temporary discharge in Table 2. Seventy-eight percent answered “the patient and family felt happiness by staying home together”; followed by “the patient and family were able to spend time peacefully (71%),” “the time spent together at home was precious (68%),” “the family were able to spend more time with the patient (66%),” “the family were satisfied of taking care of the patient at home (60%).” Overall, 22%–37% of participants reported an improvement in the patient's conditions and 15%–66% reported an improvement in the family's conditions compared to that when the patient was hospitalized.

**Table 2:** Family member's perception of the experiences of temporary discharge.

	Strong agree, agree	Unsure	Strong disagree, disagree
Family's perception about temporary discharge			
Both the patient and family felt happiness by staying at home together (*N* ​= ​87)	78	15	7
The patient and family were able to spend time peacefully (*N* = 87)	71	18	10
The time spent together at home was precious (*N* ​= ​87)	68	24	8
The family was satisfied with taking care of the patient at home (*N* ​= ​87)	60	32	8
The time spent together at home strengthened their family bond (*N* ​= ​86)	53	34	13
The family regretted having the patient leave home and be re-hospitalized (*N* ​= ​87)	26	40	33
The family felt the patient was forced to be discharged (*N* ​= ​86)	13	14	73
Patient condition compared to being hospitalized			
The patient was able to have time he/she wished to spend (*N* ​= ​87)	37	31	32
The patient had showed more smile (*N* ​= ​87)	36	41	23
The patient slept better (*N* ​= ​86)	23	41	36
The patient had increased appetite (*N* ​= ​86)	22	29	49
The patient expressed less pain (*N* ​= ​85)	22	29	48
Family condition compared to the patient being hospitalized			
The family was able to spend more time with the patient (*N* ​= ​87)	66	14	21
The family felt more burden to care for the patient (*N* ​= ​87)	46	31	23
The family felt peaceful (*N* ​= ​87)	28	39	33
The family was able to have more free time (*N* ​= ​87)	15	45	40
The family slept better (*N* ​= ​87)	15	39	46

We showed the circumstances of the patient and family caregiver before and after temporary discharge in Table 3. Regarding the circumstances of the patient and family before temporary discharge, 88% of the participants answered: “the family wanted to spend time with the patient.” Regarding the preparation for temporary discharge, 87% answered “the hospital doctor promised the patient could be re-hospitalized, if necessary.” Regarding medical support after discharge, 91% answered that, “the patient was able to be re-hospitalized on the patient's or family's request.”

**Table 3:** Circumstances of the patient and caregiver before and after temporary discharge.

	Agree	Disagree
Circumstances of the patient and family before temporary discharge		
The family wanted to spend time with the patient (*N* ​= ​86)	88	12
The patient showed obvious desire to be discharged back home (*N* ​= ​86)	81	19
The family had understood the patient would not stay long at home (*N* ​= ​85)	81	19
Pain and other symptoms were controlled (*N* ​= ​84)	80	20
The family thought the patient could be hospitalized in PCU as long as they wished (*N* ​= ​85)	65	35
The family wished to take care of the patient at home (*N* ​= ​81)	54	46
The patient needed medical treatment such as injection and drainage (*N* ​= ​86)	28	72
There was disagreement among family members about the patient's discharge (*N* ​= ​86)	6	94
Preparation of temporary discharge		
The hospital doctor promised the patient could be re-hospitalized if necessary (*N* ​= ​84)	87	13
The hospital doctor informed home visit clinics and hospital can provide consultation at any time of day (*N* ​= ​81)	85	15
The family could consult the hospital staff about daily life and home care services after being discharged (*N* ​= ​81)	79	21
The hospital doctor told the family the remaining life expectancy of the patient (*N* ​= ​85)	59	41
The family met home visit doctors and nurses before being discharged (*N* ​= ​82)	59	41
The hospital doctor strongly recommended that the patient be discharged (*N* ​= ​82)	44	56
The patient and family had a chance of staying at home overnight for trial (*N* ​= ​82)	40	60
The home visit doctor looks similar to the hospital doctor (*N* ​= ​75)	39	61
The length of time staying at home was planned in advance (*N* ​= ​84)	32	68
Medical support after discharge		
The patient was able to be re-hospitalized on the patient's or family's request (*N* ​= ​78)	91	9
Home visit doctors and nurses gave attention to the family as well (*N* ​= ​75)	84	16
The home visit clinic or PCU provided consultation at any time of day (*N* ​= ​78)	82	18
Home visit nurses had understanding of values of the patient and family (*N* ​= ​72)	81	19
Home visit doctors and nurses worked closely with the PCU staffs regarding the patient's care (*N* ​= ​72)	78	22
Home visit doctors had understanding of the values of the patient and family (*N* ​= ​71)	76	24
Home visit doctors and nurses were able to relieve the pain of the patient (*N* ​= ​72)	71	29
Home visit doctors and nurses and care manager were well-coordinated during the patient's care (*N* ​= ​73)	70	30
The patient used respite services, home help services, or volunteer services (*N* ​= ​76)	33	67

PCU, Palliative care unit.

We showed statistically significant associations between the positive experience of discharge according to the patient's opinion, and the family member's perception of the experiences of temporary discharge and circumstances of the patient and family caregiver before and after temporary discharge in Table 4. The patients who answered “the time spent together at home was precious (*P* ​= ​0.005),” “the patient was able to have time he/she wished to spend (*P* ​= ​0.02),” “the patient smiled more (*P* ​= ​0.02),” “the patient slept better (*P* ​= ​0.05),” “the patient had increased appetite (p ​= ​0.05),” “the family was able to spend more time with the patient (*P* ​= ​0.01),” “the patient showed obvious desire to be discharged back home (*P* ​= ​0.001),” “the hospital doctor promised that the patient could be re-hospitalized, if necessary (*P* ​= ​0.001),” “the hospital doctor strongly recommended that the patient be discharged (*P* ​= ​0.02),” “the patient and family had a chance of staying at home overnight for trial (*P* ​= ​0.02),” “the patient was able to be re-hospitalized on the patient's or family's request (*P* ​= ​0.008),” “home visit doctors, nurses, and care manager were well-coordinated during the patient's care (*P* ​= ​0.04),” reported more positive experience than those who did not.

**Table 4:** Associations between the positive experience of discharge according to the patient and family member's perception of the experiences of temporary discharge and circumstances of the patient and caregiver before and after temporary discharge.

	The patient expressed happiness to be discharged back home		The patient expressed regret, neither happiness nor regret, or expressed nothing		*P*-value
	*N* ​= ​63		*N* ​= ​24		
	*n*	%	*n*	%	
The time spent together at home was precious					
Strongly agree, agree	47	81.0	11	19.0	0.005
Unsure, disagree, strongly disagree	14	51.9	13	48.1	
The patient was able to have the time he/she wished to spend					
Strongly agree, agree	27	87.1	4	12.9	0.02
Unsure, disagree, strongly disagree	34	63.0	20	37.0	
The patient smiled more					
Strongly agree, agree	26	86.7	4	13.3	0.02
Unsure, disagree, strongly disagree	35	63.6	20	36.4	
The patient slept better					
Strongly agree, agree	18	90.0	2	10.0	0.05
Unsure, disagree, strongly disagree	43	67.2	21	32.8	
Patient had increased appetite					
Strongly agree, agree	22	88.0	3	12.0	0.05
Unsure, disagree, strongly disagree	38	66.7	19	33.3	
The family were able to spend more time with the patient					
Strongly agree, agree	45	80.4	11	19.6	0.01
Unsure, disagree, strongly disagree	16	55.2	13	44.8	
The patient showed obvious desire to be discharged back home					
Agree	56	81.2	13	18.8	0.001
Disagree	5	31.3	11	68.8	
The hospital doctor promised the patient could be re-hospitalized, if necessary					
Agree	56	80.0	14	20.0	0.001
Disagree	2	16.7	10	83.3	
The hospital doctor strongly recommended that the patient be discharged					
Agree	30	83.3	6	16.7	0.02
Disagree	27	70.4	18	29.6	
The patient and family had a chance to stay at home overnight for trial					
Agree	27	87.1	4	12.9	0.02
Disagree	31	62.0	19	38.0	
The patient was able to be re-hospitalized on the patient's or family's request					
Agree	53	75.7	17	24.3	0.008
Disagree	2	28.6	5	71.4	
Home visit doctors, nurses, and care manager were well-coordinated during the patient's care					
Agree	39	70.8	11	29.2	0.04
Disagree	12	54.5	10	45.5	

## Discussion

The major findings of this study are: (1) 16% of the patients who died in PCUs were discharged temporarily, (2) most of the patient and bereaved family members appreciated their experience of temporary discharge and 22%–37% reported improvement in the patient's conditions after discharge, (3) the caregivers recognized that the patient's quality of life at home was better and the hospital doctor's assurance of re-hospitalized if necessary, were strongly associate with more positive experience of discharge according to the patient's opinion than those who did not.

As for the factors related to the duration of temporary discharge, the significant variables were almost similar to those in studies that explored factors related to discharge from PCU.^17^, ^18^, ^19^ In addition, from the results of the circumstances of the patient's and caregiver's temporary discharge, most patients were in desirable conditions which are almost identical to the factors which would contribute to a home death.^20^ These results suggest that discharge planning for home death would contribute to the patient's and family's positive experience at the end-of-life regardless of the place of death.

Regarding the experience of temporary discharge, we reported the first nationwide quantitative data although clinicians have felt the benefits of temporary discharge empirically. Re-admission in the terminal stage is sometimes regarded as one of the negative quality indicators^21^; however, caregivers sometimes recognized caring for patients at home as an achievement and they do not recognize admission or re-admission and dying in the hospital negatively.^12^ These results support our recommendation for temporary discharge even if the patient cannot be expected to stay at home for a long time.

Twenty percent of patients had improved physical condition, such as pain and appetite. Although several study showed that symptom management is better in the institutional hospice setting than in homes,^22^^,^^23^ staying at home might have a positive impact on relief from physical symptoms for some patients.^16^ These benefits might result in better survival in home palliative care settings.^4^^,^^24^^,^^25^

In addition, the explanatory analysis of the factors associated with positive experience of discharge according to the patient confirmed the importance of providing appropriate home palliative after discharge and hospital doctor's assurance of re-hospitalized at the discharge counseling.

### Limitations

There are some limitations to this study. Firstly, the response rate was not very high, and we could analyze only 90 questionnaires. Secondly, the opinions of the bereaved family members might not reflect the patient's experience. However, we believe that most of the results reported by bereaved family members could be justified as 72% of the patient reported the experience of discharge to bereaved family as “I am happy to be discharged to home.” Using qualitative phenomenology design might help gain insight about the patient and the bereaved family member's experience in future studies. Thirdly, most of the questionnaire could not be validated via statistically founded methodology. Lastly, we analyzed the patients who died in PCU, and we excluded the patients whose last hospitalization was less than 3 days. We could not analyze the experience of patients and caregivers who did not die in PCU or were re-admitted at the very end-of-life.

## Conclusions

The study revealed that most patient and bereaved family members appreciated their experience of temporary discharge. The caregivers recognized that the patient's quality of life at home was better and that the hospital doctor's assurance of re-hospitalization, if necessary, was strongly associated with more positive experience of discharge by the patient than by those who did not. Appropriate home palliative care and discharge planning would contribute to a positive experience after discharge.

## Authors' contributors

Concept: Go Sekimoto, Keiko Tamura, Chieko Yamamoto; Design: Go Sekimoto, Keiko Tamura, Chieko Yamamoto. Tatsuya Morita, Yoshiyuki Kizawa, Satoru Tsuneto, Yasuo Shima, Mitsunori Miyashita; Definition of intellectual content: Go Sekimoto, Keiko Tamura, Chieko Yamamoto; Literature search: Go Sekimoto, Keiko Tamura, Chieko Yamamoto; Data acquisition: Maho Aoyama. Mitsunori Miyashita; Data analysis: Go Sekimoto, Sakiko Aso, Naoko Hayashi, Mitsunori Miyashita; Manuscript preparation: Go Sekimoto, Mitsunori Miyashita; Manuscript editing and manuscript review: Sakiko Aso, Naoko Hayashi, Keiko Tamura, Chieko Yamamoto, Maho Aoyama, Tatsuya Morita, Yoshiyuki Kizawa, Satoru Tsuneto, Yasuo Shima; Guarantor: Go Sekimoto. Mitsunori Miyashita.

## Declaration of competing of interest

None declared.
